# Optimal Use of Vasodilators for Diagnosis of Microvascular Angina in the Cardiac Catheterization Laboratory

**DOI:** 10.1161/CIRCINTERVENTIONS.120.009019

**Published:** 2020-06-10

**Authors:** Haseeb Rahman, Ozan M. Demir, Matthew Ryan, Hannah McConkey, Cian Scannell, Howard Ellis, Andrew Webb, Amedeo Chiribiri, Divaka Perera

**Affiliations:** 1 School of Cardiovascular Medicine and Sciences, British Heart Foundation Centre of Excellence and National Institute for Health Research Biomedical Research Centre (H.R., O.M.D., M.R., H.M., H.E., A.W., D.P.), King’s College London, United Kingdom.; 2 School of Biomedical Engineering and Imaging Sciences (C.S., A.C.), King’s College London, United Kingdom.

**Keywords:** coronary circulation, endothelium, magnetic resonance imaging, microvascular angina, myocardial ischemia

## Abstract

Among patients with angina and nonobstructive coronary artery disease, those with coronary microvascular dysfunction have a poor outcome. Coronary microvascular dysfunction is usually diagnosed by assessing flow reserve with an endothelium-independent vasodilator like adenosine, but the optimal diagnostic threshold is unclear. Furthermore, the incremental value of testing endothelial function has never been assessed before. We sought to determine what pharmacological thresholds correspond to exercise pathophysiology and myocardial ischemia in patients with coronary microvascular dysfunction.

**Methods:**

Patients with angina and nonobstructive coronary artery disease underwent simultaneous acquisition of coronary pressure and flow during rest, supine bicycle exercise, and pharmacological vasodilatation with adenosine and acetylcholine. Adenosine and acetylcholine coronary flow reserve were calculated as vasodilator/resting coronary blood flow (CFR and AchFR, respectively). Coronary wave intensity analysis was used to quantify the proportion of accelerating wave energy; a normal exercise response was defined as an increase in accelerating wave energy from rest to peak exercise. Ischemia was assessed by quantitative 3-Tesla stress perfusion cardiac magnetic resonance imaging and dichotomously defined by a hyperemic endo-epicardial gradient <1.0.

**Results:**

Ninety patients were enrolled (58±10 years, 77% female). Area under the curve using receiver-operating characteristic analysis demonstrated optimal CFR and AchFR thresholds for identifying exercise pathophysiology and ischemia as 2.6 and 1.5, with positive and negative predictive values of 91% and 86%, respectively. Fifty-eight percent had an abnormal CFR (of which 96% also had an abnormal AchFR). Of those with a normal CFR, 53% had an abnormal AchFR, and 47% had a normal AchFR; ischemia rates were 83%, 63%, and 14%, respectively.

**Conclusions:**

The optimal CFR and AchFR diagnostic thresholds are 2.6 and 1.5, with high-positive and negative predictive values, respectively. A normal CFR value should prompt the measurement of AchFR. A stepwise algorithm incorporating both vasodilators can accurately identify an ischemic cause in patients with nonobstructive coronary artery disease.

**What Is Known**

- Nearly half of all patients with angina are found to have unobstructed coronary arteries.
- Those with coronary microvascular dysfunction have poorer clinical outcomes; however, it is unclear how to accurately diagnose this condition in routine clinical practice.

**What the Study Adds**

- Our study has revealed that contemporary diagnostic algorithms for angina may fail to identify patients with symptoms due to coronary microvascular dysfunction.
- We propose a stepwise algorithm, with clear diagnostic thresholds incorporating tiered use of adenosine and acetylcholine pharmacological vasodilatation, validated using novel physiological tools.
- Future therapeutic studies should enroll characterized cohorts of patients with demonstrable vasodilator flow impairment assigned to medical and placebo therapies, to demonstrate the prognostic utility of these mechanistically determined thresholds.

Approximately half of all patients with angina have nonobstructive coronary artery disease (NOCAD), the majority will have occult coronary abnormalities, including coronary microvascular dysfunction (CMD), endothelial dysfunction, or coronary spasm with pharmacological vasodilators used to diagnose these entities in clinical practice.^1,2^ The most studied of these, CMD, is usually diagnosed by demonstrating impaired augmentation of coronary blood flow (CBF), or reduced coronary flow reserve (CFR), in response to adenosine. Like all biological measurements, CFR is a continuous variable but, for practical reasons, clinical algorithms and trial protocols dichotomously classify physiological indices. The COVADIS (Coronary Vasomotion Disorders International Study) group acknowledges a gray-zone, stating that CMD can be diagnosed at a CFR of below 2.0 or 2.5, a view shared by experts within the field.^3,4^ Indeed, many clinicians will only diagnose CMD and initiate therapy if the CFR is below 2.0, this dichotomy being centered around the reported incidence of death and major adverse cardiovascular events.^5–8^ Additionally, CFR only interrogates the endothelial-independent component of the coronary vasculature, as adenosine acts largely independently of endothelium. Acetylcholine interrogates the health of the endothelium, which acts as a transducer of mechanical forces (or shear-stress) and has a paracrine effect on the smooth muscle layer in the healthy heart. Acetylcholine testing in catheter laboratories is mainly confined to the diagnosis of epicardial artery vasospasm; however, graded infusion with flow assessment can characterize microvascular endothelial function and prognosticate patients with NOCAD.^9^ Coronary vasodilator testing in the catheter laboratory acts as a surrogate for abnormal coronary perfusion during physical exercise and global myocardial ischemia, but the optimal threshold of adenosine and acetylcholine-mediated flow reserve for detecting each pathophysiological state is still to be defined.^10,11^ Recent European Society of Cardiology guidelines on the management of Chronic Coronary Syndrome have strengthened the indication for coronary reactivity testing in NOCAD from IIb to IIa, and thus CMD diagnostic thresholds warrant reappraisal.^12^ The primary aim of this study was to determine the optimal CMD diagnostic threshold using adenosine-mediated CFR in patients with NOCAD, and the secondary aim was to assess the incremental value of measuring acetylcholine-mediated flow reserve (AchFR) in this cohort.

## Methods

The data that support the findings of this study are available from the corresponding author on reasonable request.

### Study Population

Consecutive patients undergoing diagnostic angiography for investigation of exertional chest pain were screened from elective waiting lists. All patients underwent adenosine based CFR assessment and a subset of patients also underwent testing with a graded intracoronary acetylcholine infusion at the discretion of the catheter laboratory operator. High-resolution perfusion cardiac magnetic resonance (CMR) was performed within 6 weeks of the index angiography procedure. Inclusion criteria were preserved left ventricular (LV) systolic function (ejection fraction >50%) and unobstructed coronary arteries (no stenosis >30% in diameter, with fractional flow reserve >0.80). Exclusion criteria were intolerance to adenosine, chronic kidney disease (estimated glomerular filtration rate <30 mL/min per m^2^), concomitant valve disease (greater than mild on echocardiography), recent acute coronary syndrome or cardiomyopathy. Antianginal medications were stopped, and patients abstained from caffeine 24 hours before all study visits. The study protocol was approved by the UK National Research Ethics Service (17/LO/0203), and all participants gave written informed consent. The study was registered with the National Institute for Health Research UK Clinical Research Network portfolio database (Central Portfolio Management System identifier: 33170).

### Catheterization Protocol

Catheterization was performed via the right radial artery using standard coronary catheters. All patients received 1 mg intravenous midazolam, 1 mg isosorbide dinitrate via the radial sheath and intraarterial unfractionated heparin (70 U/kg) before intracoronary physiological measurements. A dual pressure and Doppler sensor-tipped 0.014-inch intracoronary wire (Combowire, Volcano Philips, California) were used to measure coronary pressure and flow velocity in the left anterior descending artery, as previously described.^10^ Hemodynamic measurements were recorded under resting conditions and following intravenous adenosine-mediated hyperemia (140 μg/kg per minute) and continuously during bicycle exercise, using a specially adapted supine ergometer (Ergosana, Bitz, Germany) attached to the catheter laboratory table. Exercise began at a workload of 30 W and increased every 2 minutes by 20 W and continued until exhaustion.^10,11^ After full recovery from exercise, resting hemodynamic data was acquired before graded intracoronary acetylcholine administration for the acetylcholine study. Graded intracoronary acetylcholine concentrations of 0.182 and 18.2 µg/mL were infused (2 mL over 3 minutes) through the coronary guide catheter with cine images obtained before and after for quantitative coronary angiography.^13^ Severe coronary artery vasospasm was prespecified as >90% diameter reduction in target vessel caliber, and these patients would be excluded from subsequent analysis of coronary physiology.^14^

### Analysis of Coronary Physiological Data

Signals were sampled at 200 Hz, with data exported into a custom-made study manager program (Academic Medical Center, University of Amsterdam, the Netherlands). Pan-cardiac cycle analysis and wave intensity analysis were performed on custom-made software, Cardiac Waves (Kings College London, United Kingdom) as previously described.^10^ CFR was calculated as average peak velocity during adenosine-mediated hyperemia divided by average peak velocity during rest.

For measurement of AchFR, cross-sectional area was calculated from the coronary diameter measured 5 mm distal to the tip of the guidewire. CBF was calculated using the equation CBF = cross-sectional area × average peak velocity × 0.5 at rest (CBFrest) and following 18.2 µg/mL intracoronary acetylcholine administration (CBFach) and AchFR was calculated as (CBFach/CBFrest). We did not proceed to higher doses of provocation testing for coronary spasm in this protocol.

### Wave Intensity Analysis

Wave intensity analysis is a technique that provides directional, quantitative, and temporal information on the waves that govern coronary flow, as previously described.^10^ Perfusion efficiency is a simplified metric to indicate energy expenditure in augmentation of CBF during different physiological states and is calculated as the percentage of accelerating wave intensity in relation total wave intensity, using areas under the respective curves. In this study, change in perfusion efficiency was measured from resting condition to peak exercise; in the healthy heart perfusion efficiency has been shown to increase from rest to peak exercise, therefore, a reduction signified exercise pathophysiology.^10,11^

### 3-Tesla Perfusion CMR Imaging Protocol

All scans were performed on a dedicated 3-Tesla CMR scanner (Achieva TX, Phillips Healthcare, the Netherlands). Contiguous short-axis slices were acquired from the base to the apex for calculation of LV function and mass (CVI42, v5.1.1, Circle Cardiovascular Imaging, Calgary, ON, Canada). Following 3 minutes of intravenous adenosine (140 µg/kg per minute) stress perfusion data were acquired in 3 short-axis slices with a saturation-recovery *k-t* sensitivity encoding accelerated gradient-echo method, followed by rest perfusion 15 minutes later, using a dual-bolus gadobutrol (Gadovist, Bayer, Berlin, Germany) contrast agent scheme to correct for signal saturation of the arterial input function as previously described.^11^ Quantitative analysis was performed as previously described by Fermi-constrained deconvolution.^15^ Myocardial blood flow estimates were quantified in mL/min per gram during rest and hyperemic stress; myocardial perfusion reserve was defined as the ratio between stress and rest perfusion. A myocardial perfusion reserve <2.0 is widely accepted to signify global myocardial ischemia following vasodilator stress and was a parameter used to identify to optimal coronary vasodilator thresholds in this study.^16^ Endocardial-to-epicardial perfusion (endo/epi) ratios were calculated during hyperemic stress and rest, by comparing the inner and outer layers of myocardium averaged across the basal, mid-, and apical LV segments. The reversal of subendocardial hyperperfusion during vasodilator hyperemia is considered a marker of ischemia in patients with NOCAD.^17^ A hyperemic endo/epi ratio <1.0, signified the presence of inducible ischemia during stress and indeed forms the basis upon which visual appraisal for the presence of ischemic heart disease is performed.^18^ CMR analysis was performed by observers blinded to the catheter laboratory results.

### Statistical Analyses

The primary aim of this study was to determine the optimal CFR threshold for identifying myocardial ischemia and abnormal exercise physiology. We have adopted stress perfusion CMR as this is considered one of the most sensitive tests of ischemia that assesses the early part of the ischemic cascade and powered the study accordingly. Assuming a 50% prevalence of inducible ischemia among NOCAD patients, a sample of 75 patients gives 95% CIs of 70% to 95% for sensitivity and 63% to 92% for specificity using a CFR measurement.^19,20^ To allow for potentially unequal distribution between groups and data censoring due to quality issues and incomplete data sets, we sought to enroll 90 patients. Continuous normally distributed data are expressed as mean±SD and compared using unpaired Student *t* tests or ANOVA testing as appropriate, while categorical variables were compared with χ^2^ tests. Receiver-operating characteristic analysis was used to determine the optimal adenosine (CFR) and acetylcholine (AchFR) threshold for detecting ischemia and exercise maladaptation and likelihood ratios were used to determine optimal cutoff values. In the acetylcholine group, patients were subsequently classified based on these optimal dichotomous thresholds as concordant abnormal CFR (CFR−/AchFR−), discordant normal CFR (CFR+/AchFR−), and concordant normal CFR (CFR+/AchFR+). Correlations were assessed using the Pearson correlation coefficient with correlation coefficients displayed as ρ values. Baseline variables found to correlate with exercise perfusion efficiency or inducible ischemia on univariate analysis (*P*<0.05) were assessed by a multiple linear regression model. For all analyses, a *P* value of 0.05 was considered significant, and all *P* values were 2-sided. Statistical analyses were performed using Prism GraphPad 8.0 and SPSS version 24 (IBM Corp, Armonk, NY).

As the 2 most widely used CFR thresholds in clinical practice are 2.0 and 2.5, an exploratory analysis was also planned to compare patients with definite CMD (CFR<2.0), gray-zone (CFR, 2.0–2.5), or normal CFR (CFR>2.5) in relation to their invasive exercise physiology and CMR perfusion characteristics.

## Results

Ninety patients were enrolled into the study, 74 underwent catheter laboratory exercise, and 77 completed the CMR protocol while 40 patients additionally completed the acetylcholine study protocol. Patient characteristics are shown in Table 1. LV ejection fraction was 66±6%, LV indexed mass was 44±13 g/m^2^, and none of the subjects had scar or fibrosis identified during late gadolinium enhancement imaging. Univariate regression analysis demonstrated no effect of risk factors upon primary outcome measures of exercise coronary physiology and myocardial perfusion.

**Table 1.**
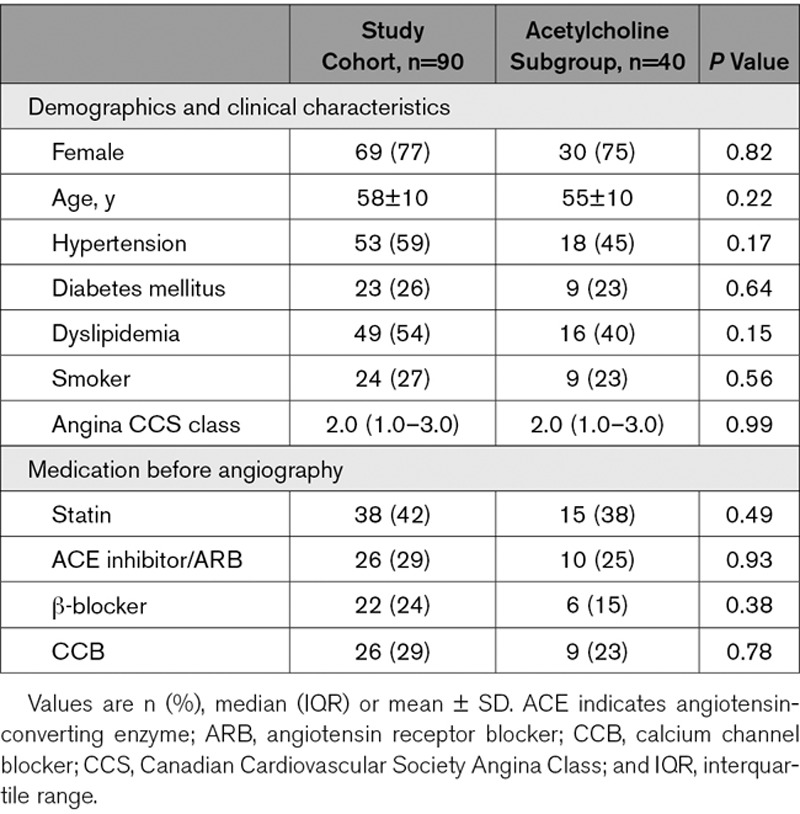
Patient Characteristics

### Optimal Vasodilator Thresholds

The optimum dichotomous CFR threshold for predicting global myocardial ischemia was 2.5 (sensitivity 95%, specificity 65%; area under the curve [AUC] =0.80, *P*<0.001) and for predicting subendocardial hypoperfusion (endo/epi<1.0) the optimal CFR value was 2.6 (sensitivity 76%, specificity 82%; AUC=0.80, *P*<0.001). The optimum dichotomous CFR threshold for predicting an improvement in exercise perfusion efficiency was 2.6 (sensitivity 83%, specificity 100%; AUC=0.91, *P*<0.001). A dichotomous CFR threshold of 2.6 had a positive predictive value of 91%, a negative predictive value of 68%, and 95% CI of 83% to 98%.

No patients had severe coronary artery vasospasm during graded infusion of acetylcholine, and thus all were included in the subsequent analysis. The optimum dichotomous AchFR value for predicting inducible ischemia was 1.5 (sensitivity 96%, specificity 54%; AUC=0.75, *P*=0.01) and for predicting an improvement in perfusion efficiency was also 1.5 (sensitivity 92%, specificity 50%; AUC=0.78, *P*=0.02). A dichotomous AchFR threshold of 1.5 had a positive predictive value of 81% and negative predictive value of 86% and 95% CI of 59% to 96%.

### Combined Vasodilator Analysis

**Figure 1.**
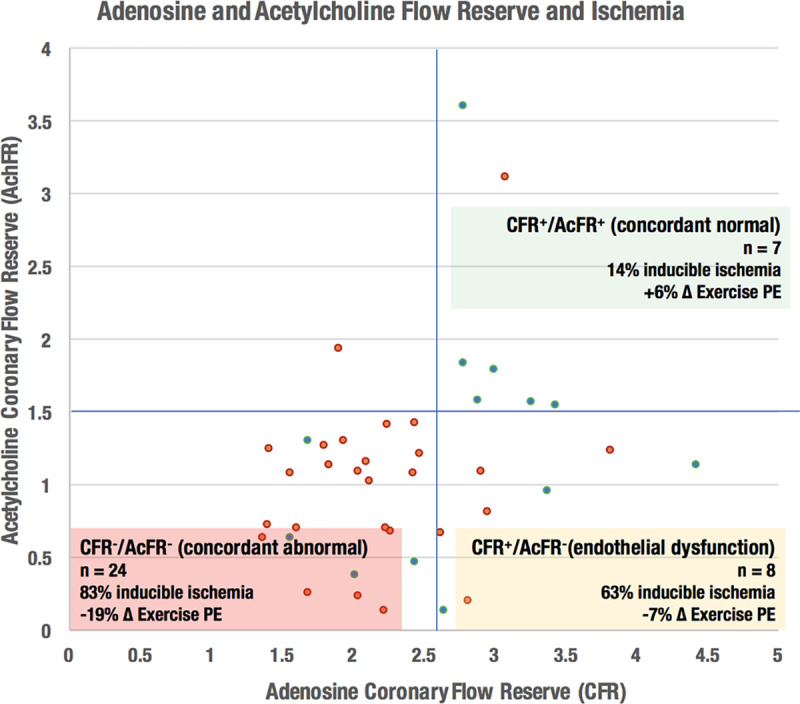
**The identification of ischemic chest pain by measurement of acetylcholine flow reserve (AchFR).** Red points signify the presence of inducible ischemia (assessed using 3-Tesla perfusion cardiac magnetic resonance imaging), while green points signify the absence of ischemia. CFR indicates coronary flow reserve; and PE, perfusion efficiency.

Applying the optimal vasodilator thresholds above, 24 patients were classified as concordant abnormal (CFR−/AchFR−), 8 as discordant normal CFR (CFR+/AchFR−) and 7 as concordant normal (CFR+/AchFR+; Figure 1). Only one patient had normal acetylcholine flow reserve despite an abnormal adenosine flow reserve. CFR−/AchFR− patients had the highest rate of inducible ischemia, followed by CFR+/AchFR− patients, while ischemia was the least common in CFR+/AchFR+ patients (83% versus 63% versus 14%). A similar pattern was observed for change in perfusion efficiency during exercise (−19% versus −7% versus +6%). Ninety-six percent (24/25) of patients with endothelial-independent dysfunction had reduced AchFR, whereas 53% (8/15) of patients with normal endothelial-independent function had reduced AchFR. Patients with CFR^+^/AchFR^−^ had a higher rate of inducible ischemia than those with normal AchFR (63% versus 14%; *P*<0.001).

### CFR Gray-Zone Analysis

A CFR threshold of <2.0 was 59% accurate at predicting global myocardial ischemia (sensitivity 41% and specificity 86%) compared with a CFR <2.5 threshold, which was 78% accurate (sensitivity 80% and specificity 76%). For predicting an improvement in perfusion efficiency on exercise, the accuracy of a CFR <2.0 threshold was 67% (sensitivity 50% and specificity 100%) compared with an accuracy of 87% for a CFR <2.5 threshold (sensitivity 81% and specificity 100%).

Myocardial perfusion and exercise physiology parameters of gray-zone patients resembled those with CMD (Table 2). The likelihood of inducible ischemia in gray-zone was 83% compared with 83% in patients with CMD (*P*=0.98) and 27% in the normal CFR group (*P*<0.001). With adenosine-mediated hyperemia, myocardial perfusion reserve was 2.66±0.42 in the normal CFR group compared with 2.00±0.36 in gray-zone and 2.01±0.48 in patients with CMD (*P*<0.001 and *P*=0.92), while the endo/epi ratio was 1.04±12 in the normal CFR group compared with 0.93±0.08 in gray-zone and 0.95±0.09 in CMD (*P*<0.001 and *P*=0.65). With exercise, coronary flow increased by 1.90±0.62 in the normal CFR group compared with 1.43±0.21 in gray-zone and 1.43±0.32 in patients with CMD (*P*=0.003 and *P*=0.96 compared with gray-zone, respectively). Perfusion efficiency during exercise was 65±14% in the normal CFR group compared with 45±8% in gray-zone and 43±12% in CMD (*P*<0.001 and *P*=0.47, respectively).

**Table 2.**
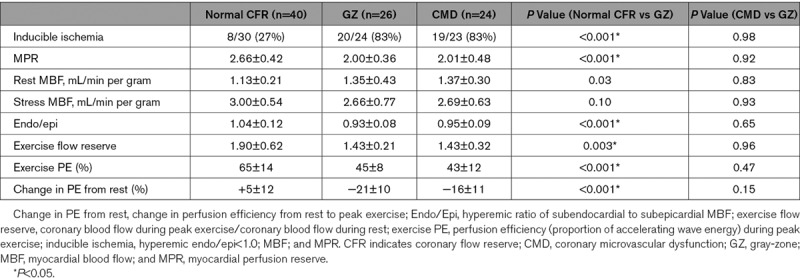
Gray-Zone Analysis of Coronary Flow Reserve

## Discussion

Combined use of vasodilator testing to stratify a NOCAD diagnosis offers the optimal accuracy for identifying abnormal exercise physiology or global myocardial ischemia and hence an ischemic substrate for chest pain. An adenosine CFR threshold of 2.6 offers excellent specificity with a high positive predictive value for ruling in ischemic chest pain while an AChFR of 1.5 has excellent sensitivity with a high negative predictive value for ruling this out. NOCAD should, therefore, first be investigated by measuring adenosine-mediated vasodilatation and if normal, acetylcholine-mediated vasodilatation; wider access to coronary flow assessment and pharmacological testing would allow improved risk stratification among this common group of patients.

### Defining the Optimal CFR

**Figure 2.**
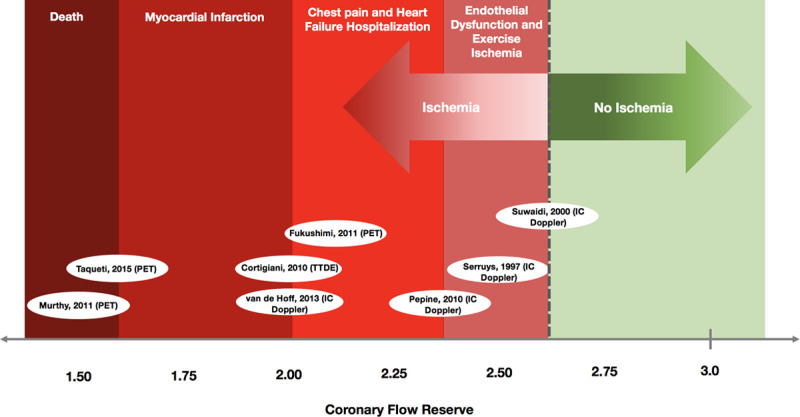
**Coronary flow reserve (CFR) and a continuum of risk.** The figure summarizes the relationship between CFR thresholds and the prognostic spectrum of cardiovascular outcomes, based on event rates from previously published studies. 3T CMR indicates 3-Tesla perfusion cardiac magnetic resonance imaging; IC Doppler, intracoronary Doppler studies; PET, position emission tomography; and TTDE, transthoracic dipyridamole echocardiography.^5–9,21–24^

Adenosine is the most widely used vasodilator in both the invasive and noninvasive setting for characterizing patients with NOCAD. The COVADIS group acknowledge a CFR gray-zone between 2.0 and 2.5 and within our unselected NOCAD cohort this encompassed nearly 30% of the study population. We have demonstrated that a dichotomous CFR threshold of 2.5 can diagnose CMD with greater accuracy than adopting a 2.0 cutoff. Previous thresholds have been defined based upon the prediction of major adverse cardiovascular events, however, the onset of ischemia and, therefore, the likelihood that a diminished CFR is better aligned with the clinical syndrome of CMD might occur earlier in the natural history of disease than the onset of death or myocardial infarctions.^5–9,21–24^ Indeed, there is a known continuous risk associated with worsening CFR and likelihood of major adverse cardiovascular events and when harder end points such as cardiovascular death are monitored, the best discriminatory CFR threshold is lower compared to prediction of angina recurrence.^25^ The continuum of risk predicted by CFR demonstrates that ischemia detection occurs before the onset of cardiovascular events, the latter perhaps too crude an end point for determining whether a patient with NOCAD has symptoms due to CMD (Figure 2). Our study has demonstrated that the onset of exercise and myocardial ischemia occurs at higher values of CFR closer to 2.6, the remaining question will be to determine whether outcome can be altered in response to earlier initiation of therapy.

### Endothelial Function Testing

As diminished endothelium-independent function is almost invariably associated with endothelial dysfunction, there would be little added benefit in measuring acetylcholine response within this group in routine clinical practice. Conversely, approximately half of all patients with normal endothelial-independent function have endothelial dysfunction, associated with a higher burden of inducible ischemia and exercise pathophysiology. Currently, the main use of acetylcholine in the catheter laboratory is for the diagnosis of coronary vasospasm. In high bolus doses, acetylcholine acts directly on muscarinic receptors in smooth muscle, producing vasoconstriction, an effect that occurs at lower doses in patients with pathological vasospastic angina.^26^ At graded infusions, in the presence of healthy endothelium, acetylcholine produces vasodilatation when administered in vivo. Hasdai et al demonstrated that patients with flow reduction to acetylcholine (endothelial dysfunction), had a greater incidence of major adverse cardiovascular events.^9^ Our study demonstrates that an increment in CBF of >50% in response to acetylcholine rules out the presence of inducible ischemia and also predicts normal exercise coronary physiology. Despite the prognostic utility of this index, acetylcholine flow reserve does not feature within international guideline criteria for the diagnosis of microvascular angina, which largely center around adenosine flow reserve measurements.^3^ Combining the high sensitivity of intracoronary acetylcholine vasodilator testing with the high specificity of adenosine testing would serve an accurate method for directly ruling out ischemic chest pain upon discovering NOCAD.

### Future Applications of Combined Vasodilator Testing

Undifferentiated NOCAD yields poor outcomes with patients often undergoing repeat invasive testing, while less sensitive noninvasive tests may fail to identify diminished CFR.^27,28^ The prevalence of microvascular dysfunction is recognized to be high among patients with NOCAD.^29^ With an increasing recommendation to treat CMD now supported by randomized-trial data, defining this condition has become increasingly paramount; while comprehensive, the COVADIS guidelines do not specify a diagnostic CFR threshold nor the role of acetylcholine vasodilator testing.^30^ Future placebo-drug trials should consider enrolling patients based upon a CFR<2.6, followed by those with AchFR<1.5, rather than adopting the historical, undifferentiated Cardiac Syndrome X definition. Use of disease-modifying therapies, such as statins and ACE (angiotensin-converting enzyme) inhibitors. may have greater benefit if initiated earlier in the disease course and should be studied in adequately powered trials enrolling well-characterized patients.^31^

**Figure 3.**
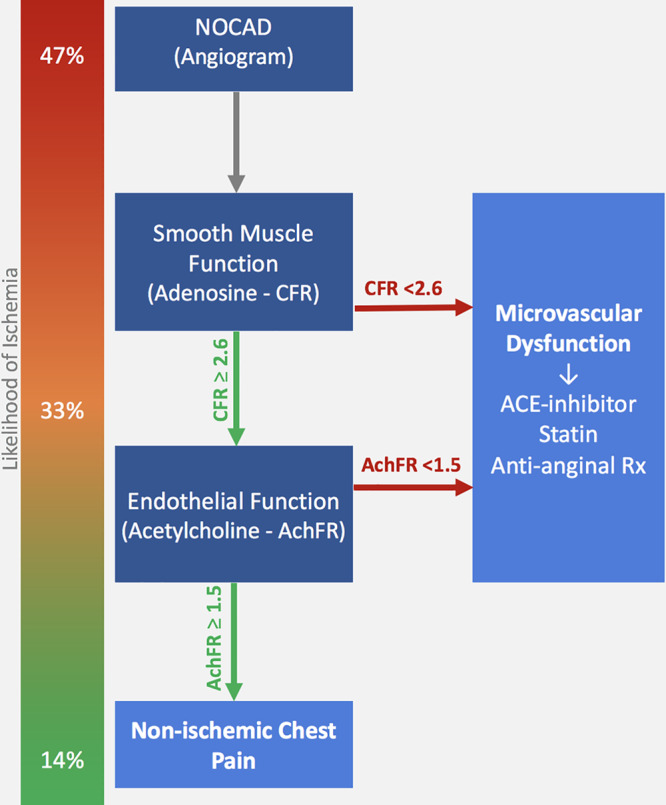
**Coronary vasodilator testing in the catheter laboratory for identifying ischemic cause of chest pain.** Likelihood of ischemia represents how progressive normal tests reduce the proportion of patients with ischemia on high-resolution cardiac magnetic resonance imaging. ACE indicates angiotensin-converting enzyme; AchFR, acetylcholine flow reserve; CFR, adenosine coronary flow reserve; and NOCAD, nonobstructive coronary artery disease.

The current study would promote the assessment of acetylcholine flow reserve, following the discovery of normal adenosine flow reserve to increase the diagnostic accuracy for ruling out an ischemic source of chest pain symptoms (Figure 3). Subsequent high-dose acetylcholine provocation testing could be performed in the same sitting, to also diagnose or exclude vasospastic angina, although this would be contingent on the wider availability of this agent within cardiac catheter laboratories. Until such time, our results indicate that the use of a single vasodilator (adenosine) would yield acceptable diagnostic accuracy and should certainly be considered above a strategy of empirical management based on angiography alone.

### Study Limitations

This was a mechanistic single-center study with relatively small numbers of patients, although this is the largest invasive exercise data set in a cohort of patients with angina and no obstructive coronary artery disease. Subendocardial hypoperfusion during hyperemia represents a very early stage of the ischemic cascade, and so its presence may not correlate perfectly with later stages such as wall motion abnormalities. Although this is a widely adopted index for identifying the presence of inducible ischemia in several clinical trials, we have also used the increasingly recognized index of myocardial perfusion reserve.^18^ Additionally, we aimed to use an invasive exercise end point in addition to help corroborate the presence of ischemia during noninvasive testing. These are surrogate markers and therapy stratified according to the onset of these changes, may not necessarily reduce the risk of major adverse cardiovascular events, and would need to be validated in adequately powered prospective studies. Our control group was not healthy volunteers but had symptoms that had led to angiography and indeed patients within this group may have abnormalities, such as coronary vasospasm, that could be unmasked during provocation testing. Our primary aim was to define the exercise physiology and myocardial perfusion of adenosine-mediated hyperemia (CFR assessment) as this is the most widely used method of characterizing CMD. Due to the demanding nature of the protocol, a smaller subgroup completed the acetylcholine study; however, this remains the largest invasive protocol with paired high-resolution perfusion imaging to date. Premedication using radial nitrates was necessary to enable bike exercise via this protocol and while the same dose was administered to each study participant, this may have attenuated the response to intracoronary acetylcholine. However, with angiography being increasingly performed via the transradial approach, this method is more representative of contemporary practice.

### Conclusions

CFR is a readily available metric following the discovery of NOCAD, capable of characterizing pathology during physical exercise and global myocardial ischemia in addition to predicting major adverse cardiovascular events. A dichotomous CFR threshold of 2.6 has an excellent positive predictive value for ruling-in the presence of ischemia; however, a normal CFR does not rule out ischemia. Subsequent measurement of AchFR using acetylcholine will have an excellent negative predictive value for ruling-out the presence of ischemia; normal response to both vasodilators would suggest a nonischemic cause of chest pain. As better characterized cohorts are enrolled into therapeutic studies, stratified management can be further refined to improve personalization of healthcare and better resource utilization.

## Sources of Funding

This work was supported by the British Heart Foundation (FS/16/49/32320 and RE/18/2/34213) and by the National Institute for Health Research via the Biomedical Research Centre award to King’s College London.

## Disclosures

None.
